# Renting royalties: How the assetization of music copyrights contributes to inequality for musicians

**DOI:** 10.1177/01634437251341715

**Published:** 2025-05-21

**Authors:** D. Bondy Valdovinos Kaye

**Affiliations:** University of Leeds, UK

**Keywords:** assets, copyright, financialization, music, musicians, royalties

## Abstract

This study presents a critique of assetization in the music industry through a case study of royalty shares and their effect on musicians. A royalty share is a form of securitized music copyright that is packaged and sold as an investment asset to buyers on royalty sharing marketplaces (RSMs). Royalty shares represent an evolutionary step in financialization of music markets that contributes to deepening inequalities by transforming copyright ownership, introducing new kinds of socio-legal obligations between musicians and financial rentiers, and shifting the way recorded music is valued. Drawing on field ethnography at music industry trade events, qualitative interviews with RSM executives, and document analysis of corporate communication and music business trade press, this article answers the question: to what extent does the rise of assetized music copyrights traded on RSMs contribute to inequality for musicians. This article argues that the assetization of musical copyrights introduces new legal frameworks to exploit musicians while maintaining existing inequalities and poor working conditions in the music industries. The conclusion reflects on the consequences of royalty shares becoming more normalized in music markets and indicates directions for future research into the assetization of cultural production.

## Introduction

A central issue at hand in recent studies of cultural production and consumption is how ongoing systemic shifts in the digital creative economy affect cultural producers. In the context of music, a “new musical system,” which is largely organized around music streaming platforms (MSPs), has been dogged by persistent expressions of public concern that streaming has made the music economy more unfair for musicians. Academic research and governmental reports complicate these notions, suggesting instead that the new musical system offers more ways for musicians to earn revenue, more avenues to distribute music independently, and more opportunities to engage with audiences. Hesmondhalgh (2021: 3610) contends that the new musical system retains the “striking inequalities and generally poor working conditions” of past configurations. If, as public critics insist, conditions *are* becoming more unfair for musicians in the new musical system, the questions are how so and why?

The objective of this article is to understand: to what extent does the rise of assetized music copyrights traded on royalty share marketplaces (RSMs) potentially contribute to inequality for musicians. RSMs are platforms that facilitate the trading of royalty shares, or the rights to future earnings from music copyrights repackaged as investment assets. Musicians can sell the rights to their future earnings to RSMs for a cash advance. Investors can bid to own portions of those future royalties by using RSM platforms. Several RSMs have emerged since 2019 alongside the proliferation of other types of financial investment into musical catalogs. These include private song management firms such as Concord, Primary Wave, and Blackstone, which have raised billions from investors to acquire the rights to music from some of the world’s most popular musical artists, living and dead. Song management firms purchase the rights to expand their private investment portfolios. These firms are not, however, the focus of this study, simply because the musical assets they acquire are generally held for private gain.

RSMs, by contrast, acquire the rights to music from a wide range of musical artists and make them available for public investment, similar to trading securities on stock markets. Corporate communication from RSMs frequently makes bold claims about the many ways selling the rights to future royalties benefits musicians. Despite these lofty ambitions, I argue that RSMs contribute to deepening inequalities by transforming copyright ownership, introducing new kinds of socio-legal obligations, and shifting the way recorded music is valued.

This article adopts a critical political economy approach to the study of cultural assets. This approach goes “beyond technical issues of efficiency to engage with basic moral questions of justice, equity and the public good” (Golding and Murdock, 2005: 61). It foregrounds historical trends and addresses legal and socio-economic factors that contribute to inequality. Using this approach, I assess the normative implications of royalty shares as they relate to musicians.

This study examines assetization in the music industry but emphasizes rentierism, as I explain in more depth below. A growing body of assetization research in science and technology studies (STS) attends to the mechanisms by which “things are turned into assets” (Birch and Muniesa, 2020). This study greatly benefits from recent work that elucidates these assetization mechanisms in the context of royalty shares (Galuszka and Legiedz, 2024). Here I build on those insights to assess the consequences of rent-seeking activity made possible by assetized music royalties. In corporate communication, RSMs emphasize the cultural value of owning musical royalty shares for investors but the espoused benefits for musicians are primarily commercial. While not inherently problematic to sell musical assets for cash-in-hand to fund further creative pursuits, I argue there are further implications of divesting partial or full ownership of musical copyrights and future royalty earnings to third party investors; a process which RSMs have vastly streamlined.

This article relies on three sources of data. I conducted ethnographic fieldwork at six international music industry trade events between April 2023 and May 2024 that featured presentations from RSM representatives. I conducted interviews with executives from RSM firms between May 2023 and September 2024, reproduced here anonymously. I collected corporate communication and press releases from the websites of four RSMs: JKBX (rebranded as ‘Jukebox’ in Februry 2025), Anote, Sonomo, and SongVest. I selected these firms because they were the largest RSMs that were actively trading royalty shares at the time of writing. Finally, I collected a total of 49 trade press articles by searching for the same four RSMs and the term “royalty shares” on six major English-language music trade press outlets: *Music Business Worldwide, MusicAlly, Billboard, Complete Music Update, and MIDiA Research.*

The remainder of the article is structured as follows. In the next section, I provide a detailed overview of royalty shares and RSMs. I then go on to distinguish music copyrights from royalty shares as two different types of IP asset. I demonstrate that trading royalty shares intensifies rent-seeking activity from music copyright assets, facilitated by RSMs and argue that this more intensified rentierism contributes to inequality. The following section argues that royalty shares and RSMs contribute to conditions of greater inequality for musicians. Royalty shares introduce new potentials for harms while also maintaining existing inequalities and poor working conditions that have historically affected musicians in the music industries. I typify seven copyright ownership arrangements that are made possible by RSMs, the different kinds of obligations introduced between musicians, RSMs, and investors, the new modes of valuation that are applied to music being sold as royalty shares, and the impact royalty shares have on musicians’ working conditions. I conclude by reflecting on the consequences of royalty shares becoming more widespread in music markets.

## The “Golden Age” of Royalty Investments

Since the blockchain boom of the mid-2010s, successive waves of music-tech startups have promised the next gold rush for music and finance. While a majority of the blockchain, Web3, and NFTs-based music startups failed to establish footholds with both investors and musicians, investment in the rights to music catalogs has steadily gained traction in the 2020s. In a 2023 guest editorial for *MusicAlly*, Anote CEO Schena (2023), claimed:

We are in a golden age for music royalties, and the good times are only going to get better. . . Artists diversifying the ways they monetize their music rights will become the norm. . .

Former JKBX CEO Scott Cohen shared this enthusiasm in a 2023 interview with *Music Business Worldwide*:

. . . when Napster came along, people in the industry viewed it as piracy, but they were dismissive of it because ultimately, why would you want some crappy mp3 download? People want the CD, they want the liner notes, they like to hold it in their hand, the tactile experience, and they love going to the record store and all that. They could never imagine that the industry would flip to what it is today. So that’s what I’m saying about music investing. We’re investing in music, and I want this to be as normalized as digital music became. . . I just hope the timeline isn’t as long. (as quoted in Ingham, 2023)

Anote, JKBX, and competing RSMs Sonomo and SongVest are financial intermediary platforms that facilitate the trading of royalty shares between musicians and investors. Royalties are revenues derived from music copyrights. There are two dominant types of music copyrights (Cooke, 2020). First are song rights pertaining to the actual musical composition. Second are recording rights, referring to the musical work fixed in some medium, such as a sound recording. Song rights are held by the musicians who composed the musical work and recording rights are generally held by the owner of the studio where the sound recordings were made. Musicians may earn royalties from one or both types of copyrights, depending on factors such as which rights are being exploited, who owns the copyrights, and who is owed fair compensation, paid on the basis of contracts between musicians and rights holders (e.g., labels and publishers).

Royalty shares are:

[S]ecurities offered by issuers on [the platform]. They represent a contractual right to receive a specified portion of royalties, fees, and other income streams contained in the income interests the issuer receives that relate to royalty rights for a specific music asset or a compilation of music assets. (JKBX, 2024)

The portion of income streams associated with royalty rights are sometimes known as fractionalized copyrights, which refers to the “practice of dividing ownership of a stream of future royalties into smaller pieces that can be traded on internet marketplaces” (Galuszka and Legiedz, 2024: 9).^1^ Similar to other types of e-commerce platforms, RSMs function differently for buyers as opposed to sellers. Buyers create accounts on RSMs, link a payment account, browse listings of royalty shares available for direct purchase or available for bidding in online auctions, and then collect earnings from royalty revenues managed and monitored via the RSM platform.

RSM FAQs and trade press covering them provide extensive, and strategically simplified, overviews of what music royalties are, how they work for buyers, and why they would make an attractive addition to investors’ portfolios. The four RSMs studied here all claim that royalty shares are “uncorrelated assets,” assets that purportedly generate revenue regardless of larger macroeconomic conditions. In press interviews, RSM executives argue that royalty shares will continue to turn profits for investors even during moments of economic downturn or turbulence because they are not connected to economic structures like federal interest rates that affect assets such as real estate. In an interview one RSM executive explained:

There is an assumption that there are lots of asset classes that are uncorrelated. Most of the time this is not true. Most asset classes depend on how the real economy is behaving. In the music industry you have this pattern that is kind of broken. You don’t really have a correlation with the real economy or with consumer spending. The main risk factor when you invest in music is you are investing in listening habits. You are betting that people will keep listening to the same songs over and over again. (personal communication 2024)

Music copyrights will continue to generate royalties as long as people continue to purchase or stream music, which is often a factor of their popularity. In the new musical system, royalty generation is heavily reliant on artists, songs, and catalog being recommended to listeners by algorithmic systems on MSPs. This likelihood to be recommended is one of many factors that contribute to new ways music is valued (as I discuss further below).

**Figure 1.**
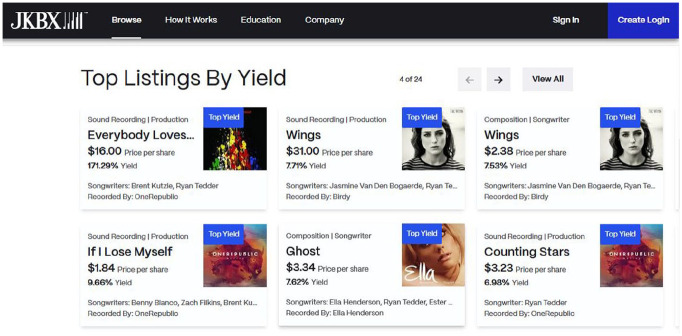
JKBX royalty share listings, accessed November 2024.

**Figure 2.**
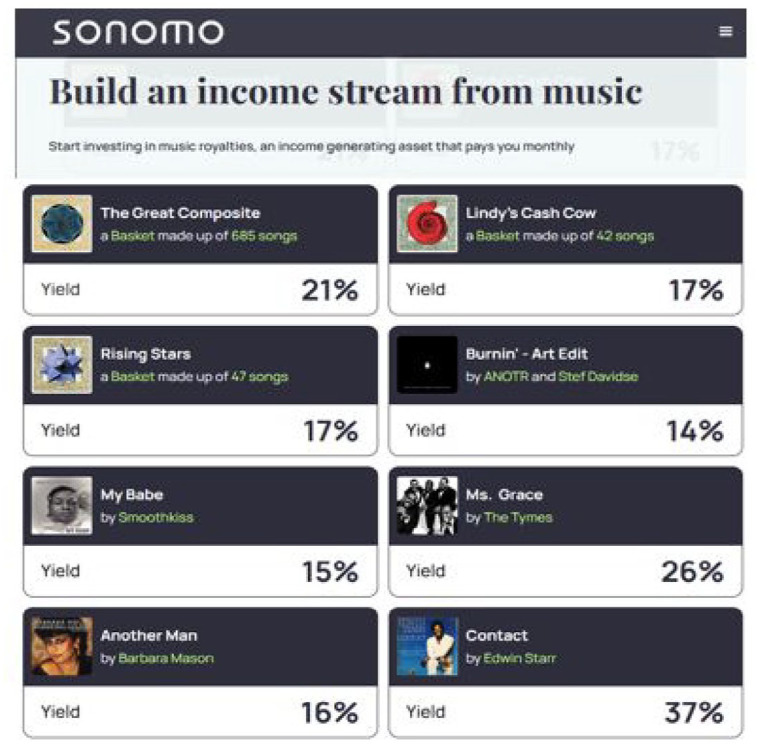
Sonomo royalty share listings, accessed November 2024.

**Figure 3.**
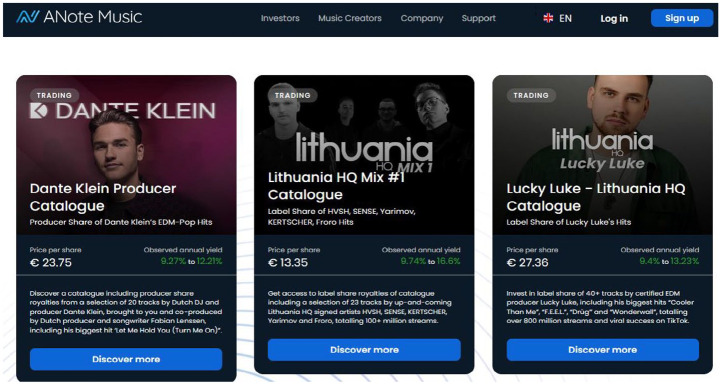
Anote royalty share listings, accessed November 2024.

Moving on to how RSMs function for sellers, musicians and their management teams provide the actual assets being sold on these platforms. The contractual terms offered to musicians to sell royalty shares differ by RSM. Musicians can sell the rights to sound recordings and/or compositions (Figure 1, JKBX), individual songs and/or bundles of songs (Figure 2, Sonomo), and entire catalogs (Figure 3, Anote).

The FAQ and informational pages on RSM websites provide overviews of what royalty shares are, how they work, and what kinds of benefits they offer to musicians who choose to sell their royalties. The most obvious and immediate benefits are the upfront payments that musicians receive from successful royalty share sales. Other benefits highlighted include maintaining control over certain rights, reverting control of rights back to musicians after a period of time passes, and flexible offerings. The exact terms of sale agreements are often bespoke and negotiated between the platform and the musician’s management team (RSM executive, personal communication 2024). The following are Anote’s (2024) agreement template options as listed on their website as of November 2024:

- Permanent listing: give access to your music catalogs’ royalty streams in perpetuity (lifetime of artist + 70 years after death) to receive a higher valuation
- Temporary listing: see full ownership return to you at the end of the chosen deal term (e.g., 5, 10, or 12 years), ideal to re-evaluate your catalogs’ status in a few years’ time
- Include future creations: your back catalog provides known data, while additional future creations may result in further success. Choose to include future creations to boost growth opportunities.
- Buyback compatibility: stay ready for future opportunities and see ownership instantly return. Including this option allows you to purchase your rights back with a small premium at any point.

Notably, the most profitable listing option is for musicians to sell away rights to full catalogs to royalty share investors in perpetuity. Musicians must also choose to opt in to buyback agreements that include fees to reclaim rights.

Selling royalty shares is not a new phenomenon. In the late 1990s, David Bowie, working alongside an American investment banker, leveraged the value of the artist’s musical catalog to create a musical asset that would come to be known as “Bowie Bonds.” The bonds were issued in 1997 to American insurance company Prudential Insurance for $55million (USD) with a maturity length of 10 years. Bowie used the capital to buy back his master recordings from his former label RCA Records and to finance his next album projects. Once the bonds reached maturity, the rights reverted back to Bowie (Christman, 2016). The landscape of the Anglo-American music market shifted in the 2000s. Revenues from recorded music were in decline and a global financial crisis was looming on the horizon. Despite interest in replicating Bowie Bonds, tech entrepreneur Sean Peace explained the marketplace for royalty shares was not ideal to attract investors in 2007 when he initially launched SongVest. Peace relaunched the company in 2020 at a time when, in his view, music market conditions and financial securities policies combined to make conditions much more favorable for royalty investment (ArtistConnect, 2023).

**Figure 4.**
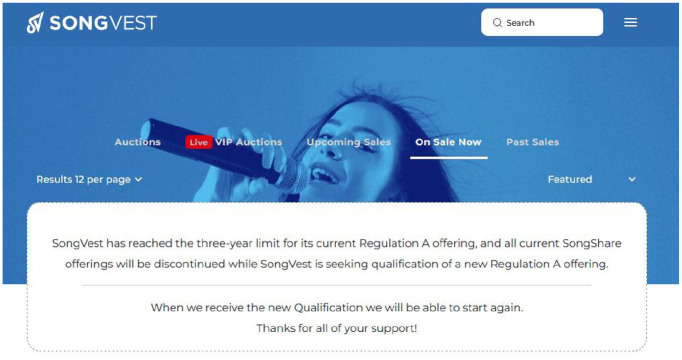
SongVest SEC limit announcement, accessed November 2024.

Regulation of royalty shares is still in its infancy. US-based RSMs SongVest and JKBX both offer royalty shares that are guaranteed by the US Securities and Exchange Commission (SEC). In separate press interviews, former JKBX CEO Scott Cohen (Ingham, 2023) and SongVest CEO Sean Peace (ArtistConnect, 2023) stated that having SEC qualification adds legitimacy to their offerings to reassure both investors and musicians.^2^ The regulatory approval process is time consuming. JKBX had to delay their public launch for several months in 2023 while waiting for approval. SEC approval also carries certain restrictions. In November 2024, SongVest announced it could no longer offer royalty shares after reaching its SEC mandated 3-year limit and would have to pause trading until it received another qualification (Figure 4).

Global market growth and financialization has primed the music market for the dramatic resurgence of royalty shares. Between 2014 and 2023, the total value of music copyright grew from US $24bn to US $45.5bn (Page, 2023). In the past two decades the music industries have experienced intensive financialization characterized by factors such as more concentrated corporate ownership, the entry of finance capital and private equity firms into music markets, and the emergence of song management funds (deWaard, 2024). Galuszka and Legiedz (2024) point to RSMs as further evidence of the ongoing financialization of the music industries. Andrew deWaard (2024: 100) argues that the financialization of music has been “lucrative for corporations and superstar musicians, but devastating for average musicians. Inequality and exploitation are rampant.” A financialized music market generates profits not only from real production of new music but through financial activity; namely collecting new forms of rents from musical assets.

## Rent-Bearing Copyrights and Rent-Seeking Royalty Shares

Music copyrights and by extension royalty shares generate profit by extracting financial rents. Economic geographer Brett Christophers (2020) draws on two schools of economic thought to construct a definition of rent. First is the heterodox economic understanding that conceives of rent as being asset-based income that is concerned with extracting value rather than creating value. As Christophers (2020: 22) explains, certain types of assets are particularly well-suited for value extraction, and intellectual property rights (of which music copyrights form a distinct example) are one. Rent according to this view is parasitic; designing and perpetuating extractive conditions to derive profit. Second is the orthodox economic view that focuses on how markets behave under conditions of competition. Rather than focusing on the assets involved in generating rent or specific actions of rentiers, this view attributes rentier profits to abnormal market conditions, such as highly concentrated monopolistic or oligopolistic markets. Merging these viewpoints, Christophers (2020) arrives at his definition of rent as “income derived from the ownership, possession or control of scarce assets under conditions of limited or no competition” (p. 28).

There is an important distinction between rent-bearing assets and rent-seeking activity in the context of music copyrights, which Christophers does *not* discuss in his book. Music copyrights are *rent-bearing* assets, meaning they generate money for owners, or rightsholders. Like other forms of commercial activity, rent-bearing assets generate more profit through commercial exploitation and under conditions of market concentration. Royalty shares allow for more aggressive *rent-seeking* that focuses on extracting greater value from existing IP assets without investing profits into the creation of new IP. Musicians can sell their copyrights to RSMs through a variety of different schemes and those shares are then traded, via RSMs, by retail investors. This process does not directly entail new music being created. However, one way that RSMs market their services to musicians is by highlighting the ways they can use the capital earned from royalty share sales to fund future creative endeavors, just as David Bowie did in the late 1990s.

Rent-bearing assets are “characterized by monopoly power not just in ownership or control – the heterodox emphasis – but also in terms of their commercialization on the market” (Christophers, 2020: 28). Rent-seeking activity “entails a particular mode of rentierism: investing time and money more in sweating existing rent-generating assets than in carrying out the research and development necessary to create new [assets]” (Christophers, 2020: 193). While “arguably, all rentiers have such a [rent-seeking] tendency” Christophers (2020: 193) asserts, “some display it much more emphatically than others, not least, in recent times, IP rentiers.” IP markets adhere to norms and conditions that both enable, reward, and incentivize rent-seeking activity including IP policy that favors large (western) corporate entities, (de)regulation of specific IP markets, and a tendency toward oligopolistic structures. A prime example of this tendency is the Trade Related Aspects of Intellectual Property (TRIPS) Agreement that sets minimum requirements for IP protections to which all members of the World Trade Organization must adhere. TRIPS creates a deeply inequitable paradigm that seeks to “extend western models of intellectual property law that are deeply discordant with development policies and strategies” (Drahos, 2016: 8). Legal scholar Valbona Muzaka (2013) describes TRIPS as one of the most significant pieces of trade policy in contemporary history that sets high standards of protection and enforcement that apply across national contexts regardless of social, economic, legal, or political conditions.

To be clear, rent-seeking activity has always been a core feature of music industries. Royalty shares and RSMs represent a move toward more aggressive rent-seeking activity, or what Christophers (2020) refers to as rentierization. The proliferation of rentierization goes hand-in-hand with processes of financialization, that are demonstrably shifting music markets further into financial markets and injecting financial logics into cultural industries (deWaard, 2024). A rentier system is designed such that “gains flow disproportionately to the small handful of individuals who create, capture or protect from competition or impairment the all-important rent-generating assets” (Christophers, 2020: 76). Rentiers seek to invent new kinds of financial instruments that extract more value from rent-bearing assets. Contractual agreements that split music copyrights into smaller constituent fractions and platform marketplaces to buy and sell those copyrights represent an interesting example of this. A highly renterized market is one in which returns from rent-bearing assets exceed growth. According to Christophers (2020: 82), a rentier economy represents the materialization of perfect conditions to produce inequality.

## Are Royalty Shares Making Conditions More Unequal for Musicians?

The recent royalty shares gold rush reflects the culmination of rentierization and financialization in music markets in the years since the debut of Bowie Bonds. For more than two decades financial intermediaries, like SongVest originally founded in 2007, have been poised to capitalize on the rent-seeking potential of royalty shares. Now that the gold rush has begun, it is vital to examine the consequences of rentierization in the music market as experienced by those making music.

### Ownership

**Figure 5.**
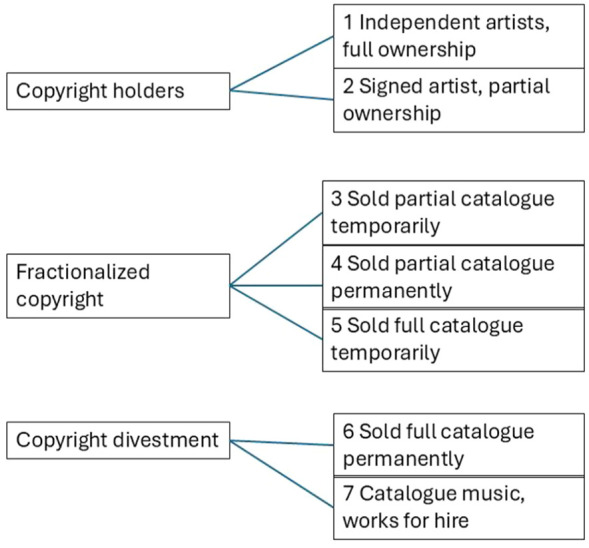
Taxonomy of copyright ownership facilitated by RSMs. Source: author.

Selling royalty shares introduces new kinds of copyright ownership arrangements for musicians. Transformation of ownership is a key driver of social inequality fomented by rentierization (Christophers, 2020: 392). Christophers explores how rentier markets seek to concentrate ownership of assets and, thereby, wealth. In this context, RSMs serve as the financial instrument by which musicians may transfer copyright ownership, partially or entirely, to third party investors. Figure 5 below depicts seven types of copyright ownership arrangements facilitated by RSMs.^3^ There are, of course, traditional arrangements commonly found across music industries including independent artists who fully own their music copyrights (position 1), signed artists who assign certain rights to their label or publisher (position 2), and artists who intentionally sell their music copyrights to be used as library/catalog music or according to certain works-for-hire contracts (position 7). Positions 3–6 entail rent-seeking royalty share arrangements facilitated by RSMs.^4^ As detailed above, RSMs offer many different royalty share agreements to musicians.

Positions 3–5 involve fractionalized copyright transfers. Using Anote’s agreement templates as a reference point, musicians may sell the rights to certain songs, albums, or bundles, while retaining control of the rest of their catalog and see their rights returned to them after a period of time has elapsed (position 3). Anote’s corporate communication underscores that selling rights permanently will result in higher payouts to entice musicians to select a more irreversible, more dispossessed, ownership position (positions 4 and 6). The immediate benefits of a higher sale price for musicians are shorter-term as compared to the longer-term benefits for investors collecting earnings from royalties for the entire duration of the copyright.

Selling entire catalogs (positions 5 and 6) involves lengthier negotiations and results in even more valuable offerings. As reported in press, these are the kinds of agreements that JKBX pursues with its clients. It is worth noting that selling “full catalogs” does not necessarily mean selling away all of one’s music copyrights. To be sure, musicians *can* sell off all of their copyrights, but publicly available royalty share agreements suggest more often musicians will sell certain rights while retaining others, such as composition rights but not sound recording rights or vice versa (see Figure 1).

Royalty share sales result in debt-free payments, meaning musicians do not need to pay back a creditor, unlike traditional advances offered by record labels. Corporate communication from RSMs often asserts that selling royalty shares gives musicians *more* control of their music. As one executive explained:

If an independent label is selling 2% of their catalogue it’s not because they want to cash out. It’s because they’re trying to get some money to reinvest in the production of music. There is a flow of this money back into production. . . an individual artist might be trying to gather some money to invest in their own production so they can be more independent. (personal communication, 2024).

RSMs market themselves as services that offer financial opportunities to musicians by granting them access to fast capital and a way for musicians to wrest control away from record labels or publishers. However, RSMs are not reinvesting capital in new IP production as a record label would. They offer control through capital at the cost of ownership. Their value proposal entails swapping a contractual relationship with a music company for a contractual relationship with a financial intermediary.

### Obligation

Royalty shares introduce new kinds of obligations between musicians, financial intermediaries, and investors that are “legally enforceable” and “emotionally fortified” (Tellmann, 2022: 34). Legally enforceable obligations in the music industries are formalized through recording and publishing contracts. These contracts have long served as legal mechanisms of obligation between musicians and record labels, publishers, distributors, and other musical intermediaries. Cultural labor scholar Stahl (2012) applies the concept of contractarianism, drawing on the work of feminist legal scholar Carol Pateman, to analyze the relationship between musicians who enjoy control over the copyrights to their works but are beholden to corporate entities according to terms laid out in contractual agreements with labels or publishers. Historically speaking, these terms are generally more favorable for superstars than emerging artists.

The royalty share agreements examined here are voluntary and consensual. Musicians either approach or are approached by RSMs to sell their royalties. Musicians and their management teams have the final say when deciding to enter into royalty sharing agreements. There are no compulsory mechanisms, as yet, but contractual consent is historically murky in the music business. As Stahl (2012: 178–179) argues, “when record companies finally stop cheerleading, advising, and counseling and start commanding, their actions can strike the artist as coercive.” Given that RSM executives hope to see royalty share trading become as commonplace as digital music in the music economy, advocates, policymakers, and researchers must remain vigilant as royalty share agreements options become more widespread and potentially more coercive. There are also important overlooked questions about assetizing income from copyright royalties. Income from copyright royalties has historically made up a minority proportion of income for a majority of musicians, which appears to hold true in the streaming era (see e.g., Hesmondhalgh et al., 2021).

Once a royalty share sale is finalized, musicians are under no legal obligation to ensure their assets perform as forecasted for investors. Yet, there may still be emotionally fortified obligations linking together musicians and certain investors, namely fans. Internet scholar Nancy Baym (2018: 81) distinguishes fans from other kinds of music listeners according to their “level of feeling invested in the object of their fandom and the kinds of practices in which they engage.” Some RSMs explicitly seek to leverage the affective relationships between musicians and fans. Around the time of its originally planned public launch in 2023, JKBX hedged bets on fans’ willingness to invest “billions” in music royalty shares (Marshall, 2023). The following year, JKBX announced it was partnering with fan-funding platform Corite to “open up JKBX’s platform to Corite’s audience” (Dredge, 2024). SongVest expressly markets itself as a “musical memorabilia” service aimed encouraging music fans to become invested fans:

Earn bragging rights when you tell your friends you invested in your favorite favorite music [sic]. There are only a limited supply of SongShares® made available for purchase, so everyone who holds SongShares is part of a community that directly supports artists and songwriters. And because earned royalties are paid out quarterly, you have an ongoing relationship with your favorite songs and artists. (SongVest, 2024)

Baym (2018: 82) highlights tensions between fans’ desire to reject norms of capitalism and their willingness to embrace consumerism. She points to examples of fans investing in cultural artifacts with little or no economic value. This suggests that fans may very well be motivated to invest in royalty shares that are forecasted to earn them returns on their investment. RSMs state in press, corporate communication, and purchasing agreements that royalty shares are risky investments and may not live up to their forecasted earning potential; in other words, buyers beware. This introduces the risk of reputational damage to musicians who sell shares that do not perform as expected. Perhaps more concerningly, it also enables financial exploitation that preys on fans’ affective relationships with their favorite artists.

### Value

Royalty shares influence the way recorded music is valued. Formally quantifying the value of intangible assets is a lengthy and complicated process often involving a mélange of in-house technical experts, financial intermediaries, and public regulators (Chiapello, 2015). Appraising “hard to value” cultural assets involves additional methods and instruments to assess non-monetary value (Lamont, 2012; Plante et al., 2021). The value of cultural assets is contingent on “uniqueness, authenticity, particularity, and specialty” (Harvey, 2002: 103). The value of cultural goods can be influenced by quantitative factors, such as historical pricing and public market data, or qualitative factors that are more opaque, such as taste, aesthetics, and artistic appraisal by cultural critics (Coslor, 2016). When presented with complex and overwhelming financial data, investors may defer to more imprecise means of determining value, such as simple rule of thumb (Coslor, 2016). As Scott Cohen acknowledges in a 2023 interview with *Forbes*:

Some investors may not understand things like price to earnings ratios or what drives a tech stock like Google, Apple or Amazon, but when you put music in front of them, they understand what songs are valuable and why they are valuable. (as quoted in Hochberg, 2023)

The implication of this claim is that royalty share investors are more likely to understand why music is more valuable than other types of assets. But is this the case? Cohen makes the assumption that investors buying royalty shares are familiar with music. It is fair to assume most investors would know big names like Adele or Beyoncé, but perhaps not their songwriters, whose names are often hidden away in recording credits. Investors are even less likely to have heard of young, obscure, or emerging artists, which is precisely why JKBX seeks to purchase and list rights to only the biggest names in music. Royalty investors who are not familiar with music would be no more familiar with how future musical value is calculated by RSMs than they would be to understand earning ratios that drive tech stocks. In those instances, investors rely on the estimated annual yield that is calculated and displayed by the RSM storefront (see Figures 1–3 above).

The exact methods RSMs use to calculate value are proprietary, but RSM FAQs indicate that RSMs draw heavily on quantitative data from MSPs. Musicians must meet a minimum number of streams per month over a set duration of time to be eligible to sell their royalty shares on RSMs.^5^ Relying on metrics to value music connects to wider problems with streaming metrics such as the potential for error, misattribution, or data loss associated with a lack of data standardization across the music industries (see Campos Valverde, 2025, forthcoming). It also ties to the ways in which MSP algorithmic recommender systems reproduce conditions of inequality for musicians (Hesmondhalgh et al., 2023). Moreover, the business of music, like other cultural industries, is fundamentally risky; it is challenging, if not impossible, to predict the how popular music will be in the future based on metrics of past performance.

RSMs make *recommendations* to clients based on their evaluations and then let the selling party (musician, manager, lawyer, etc.) decide on the final listing price:

We support the determination of the catalogue’s valuation and you decide your ideal auction target price. A fair starting valuation is important for both the seller and for the buyers. (Anote, 2024)

The reserve price and minimum bid will be mutually determined by you and SongVest management. We want to give you complete control of your listings, as well as provide your auction with the best possible probability of success. (SongVest, 2024)

Your tracks get suggested valuations based on their streaming performance. Sell your tracks’ future royalties based on desired valuations. (Sonomo, 2024)

These statements are made to reassure musicians that they remain in control of how their music is valued, but still strongly imply that the royalty shares must sell. If shares do not sell, control reverts back to the musician to try again in another sale or withdraw from selling. This creates a potential tension point between the financial intermediary and the musicians selling their rights. If royalty shares do not sell at an agreed-upon price point, musicians may agree to adjust prices to what the RSM advises will be more likely to sell.

RSMs make money through transaction costs, administrative fees, and buyback fees. JKBX’s SEC offering circular indicates that it collects income from purchase agreements, fees associated with servicing royalty shares, and 1% interest on gross monies earned (JKBX, 2023). SongVest’s SEC offering circular (SEC, 2023) notes that payments from royalty share sales cover the actual cost of the shares sold, offering expenses such as legal, accounting, escrow, and compliance costs, acquisition expenses such as due diligence and legal costs, and sourcing fees between 0% and 25% that cover the cost of acquiring royalties to sell as shares. Additionally, as shown above, Anote’s (2024) agreement templates include a buyback option for musicians to purchase their rights for a “small premium.”

RSMs repeatedly make claims that their services maximize value for musicians. In an interview one RSM executive suggested that musicians were likely to *undervalue* their music, which would ultimately benefit them financially when their royalty shares were sold at auction:

We have observed that the valuations at which catalogues are being sold on our platforms are a little bit higher than before when they were being sold on private markets. There are fewer players involved in the negotiations and there is more power asymmetry. The valuations and prices are more efficient and more skewed towards the party that has more leverage. . . more money. When you introduce an auction model like we do, where multiple players can have a say in the value of the catalogue, you typically have higher prices for the sellers and lower yield for investors. The result from our experience is that the introduction of a public market approach for the music industry is devaluation of catalogues, decreasing the yield for the investors. This is called in finance decreasing the cost of capital. Put in more generic terms we are reducing the cost of capital in the music industry. It costs you less to borrow money. (personal communication 2024)

That RSMs are reducing the cost of capital in the music industry relies on the assumption that raw auction prices are a reliable measure of value for cultural assets, despite previous research that contends auction prices for art are volatile, opaque, and often require more nuanced interpretations from experts (Coslor, 2016). Hard-to-value cultural goods like music copyrights add further complexities to auctions in which buyers will be taking the unique characteristics of the assets being sold into consideration when making decisions that affect sellers. A more thorough unpacking of the claim that RSMs are devaluing the cost of capital in the music market is beyond the scope of this study but speaks to the view widely shared among these firms that selling away royalties actually benefits musicians.

Turning future incomes into assets can be understood as value-enhancing for the seller only if they are motivated by entrepreneurial mindsets, seeking to maximize value and overall financial rewards from their work (Cooper, 2015). Previous studies of the working lives of musicians suggest that some musicians might adhere to these entrepreneurial mindsets, but they are often not driven *solely* by commercial motivations (e.g., Everts et al., 2022; Haynes and Marshall, 2018).

### Working Conditions

Why would musicians agree to sell their royalties? The obvious answer is the quick and debt-free payout from a successful sale. As Sonomo (2024) entreats, “stop waiting years for your royalties to add up to a meaningful sum. . . get the full amount today.” The question is whether obtaining that full amount upfront makes working conditions better or worse for musicians in the longer term. Nappert and Plante (2023) present an illuminating case study of minor league baseball players equitizing their future earnings to be sold as investment, in a manner similar to musicians selling royalty shares. A key difference between equitizing income and selling royalty shares is that copyright royalties are only one source of income for musicians, whereas minor league players in Nappert and Plante’s study were equitizing their primary income. Even still, there are salient parallels between their study of baseball players and this study of musicians. Equitizing income, Nappert and Plante explain, can be a way to escape poverty for early-career minor league baseball players.^6^ Equitization models are sold to players as a way to invest in their future success and capitalize on their talent, and these models privilege high-earning players.

Low wages and undervaluing skilled labor are indisputably issues that plague musicians, particularly those outside the echelons of superstardom. JKBX’s business model, as the main exception, show how royalty share markets prioritize popular high-earning musicians over lesser known or emerging artists. Yet, the pool of superstars is more limited and to acquire these valuable right JKBX must compete against heavyweight private equity firms and song management funds. For emerging artists in financially weaker positions, royalty shares may be a particularly appealing means to make a quick payday at the expense of future copyright earnings. Corporate communication from RSMs echoes the language of hope that trains focus on longer term benefits musicians reap from future career returns (Mouritsen and Kreiner, 2016). In the context of minor league baseball, Nappert and Plante (2023) raise crucial questions about financial literacy among players who enter into equitization agreements as another factor that contributes to inequality.

The proliferation of RSMs creates further incentive structures to maintain poor working conditions. According to Nappert and Plante (2023: 17):

. . . one could argue that the whole equitization model depends on low wages being sustained. If all minor league players earned decent wages, the need for equitization, at least to escape poverty, would be greatly diminished. This suggests that assetization is more likely to become a popular phenomenon in contexts where skilled labour is undervalued. . .

RSMs take advantage of the longstanding unstable working conditions in music industries for more aggressive rent-seeking. Royalty sharing empowers financial rentiers and disempowers cultural producers. Stahl (2012) argues that self-determination for musicians is derived, in part, from legal controls over their creative outputs that is afforded to them by legal structures like copyright. He explains how a key aspect of financialization is stripping away those controls through legally enforceable instruments that assign away copyrights and subordinate musicians. RSMs do indeed represent a deeper descent into a financialized music market (Galuszka and Legiedz, 2024) that do not alleviate existing problems with musicians’ working conditions while opening new legal manifolds for exploitation through rentierization.

## Concluding Thoughts

Royalty shares contribute to inequality in the new musical system by enabling more aggressive rent-seeking activity from music copyrights that primarily benefits financial rentiers and not (most) musicians. They capitalize on the excessive rent-seeking potential of intellectual property rights that pushes beyond what is necessary or should be politically tolerable (Harvey, 2019). Evidence suggests that in the streaming era the vast majority of musicians still struggle to earn a sustainable living from recorded music (Hesmondhalgh et al., 2021). RSMs present themselves as an alternative to labels for musicians to access cash advances, offering fast capital at the cost of future earnings and ownership. Selling royalty shares does not ameliorate income inequality issues and may, in fact, exacerbate wealth inequality among musicians.

The economics of royalty share sales favors superstar musicians. JKBX is well-aware of this. They have designed a business model to capitalize on the value of highly popular songs from well-known superstars and songwriters. For RSMs working with lesser known or emerging artists, the potential for exploitation is greater. Low wages and unstable working conditions mean that non-superstar musicians are likely to be in need of a quick cash infusion. A lack of financial expertise may mean they are likely to accept the sale price offered to them by an RSM, calculated using dubious MSP data. In this regard, MSPs *do* contribute to making conditions more unfair for musicians through the role they play in assigning value to musical assets traded on RSMs.

Four directions for future research follow on from this study. First is policy and regulation. Future research in this area should explore how regulation shapes asset economies, how secondary asset markets are regulated, and what kinds of contractual issues will be raised when disputes emerge between sellers, investors, and intermediaries. Second, future studies need to explore the expansion and effects of cultural rentierism in global majority contexts. This study has overly focused on an Anglo-American cultural context to account for four RSMs that are incorporated and mainly operate in the US, UK, and Europe. Third, critical research must not overlook or discount the agency of musicians when debating the fairness of royalty shares. Empirical research with musicians and music managers who elect to sell their music copyrights as royalty shares is needed to understand more about their motivations and what happens when tensions arise. Finally, while this study focused specifically on musicians, it is important for scholars of media and cultural industries to engage with these assetization debates in other cultural domains. The arrival of new investment platforms like Investable (formerly known as LabelCoin) suggests music is being used as a test case for new iterations of RSMs pitching themselves to investors as hubs for bidding on cultural assets such as film, literature, and sports.

Improving conditions for cultural producers throughout the digital creative economy requires diagnosing the institutional and legal structures that contribute to inequality as part of the search for more just alternatives. Christophers (2020: 394) finds hope in the public sector returning to own and operate those key assets that undergird social life; music copyrights in this context. A more just system of cultural production would be supported by grants and public funding rather than relying on infusions of capital from RSMs that come at the cost of future royalty earnings and copyright ownership. In the interim, if royalty shares indeed become a fixture of new musical system, knowledge pooling and informational resources are needed to ensure musicians are aware of the terms of contractual agreements for royalty sharing. Regulatory oversight must monitor these markets as they mature and expand into other cultural domains. Like musicians, other digital cultural producers must critically interrogate claims about the transformative benefits of selling away future earnings that belie the exploitative and extractive nature of assetization.

## Notes

1. See Galuszka and Legiedz (2024) for a more comprehensive overview of the actual process by which music copyrights are turned into royalty share assets and made available for sale.

2. A more rigorous analysis of the JKBX and SongVest’s SEC regulation-A qualifications is beyond the scope of this article.

3. This model is used for illustrative purposes only. See Galuszka and Legiedz (2024) for a more detailed breakdown of the different kinds of music copyrights that can be sold as royalty shares.

4. These four positions, particularly position 6, are also made possibly by selling catalogs to song management funds. For the purposes of scope, my analysis is limited to RSMs.

5. One outlier among the four RSMs analyzed here is JKBX that mainly approaches large superstar artists to broker deals for listings.

6. Nappert and Plante cite a report that indicates the average minor league baseball player makes under US $15,000 annually. See Fagan (2021).
